# A sport-for-protection program reduces suicidal ideation in youth affected by displacement: a secondary analysis of the Game Connect trial in Uganda

**DOI:** 10.3389/fpsyt.2025.1569793

**Published:** 2025-07-04

**Authors:** Kathleen Latimer, Rita Larok, John Paul Nyeko, Bashir Lukungu, Simon Rosenbaum, Lydia Murungi, Robinah Nannungi, Jeroen Carrin, Esther Nanfuka Kalule, Ronald Luwangula, Davy Vancampfort

**Affiliations:** ^1^ Olympic Refuge Foundation, Lausanne, Switzerland; ^2^ Association of Volunteers in International Service (AVSI), Kampala, Uganda; ^3^ International Health Sciences University, Kampala, Uganda; ^4^ Institute of Statistics and Applied Economics, Makerere University, Kampala, Uganda; ^5^ Discipline of Psychiatry and Mental Health, School of Clinical Medicine, University of New South Wales, Sydney, NSW, Australia; ^6^ Olympic Refuge Foundation Think Tank, Lausanne, Switzerland; ^7^ Department of Social Work and Social Administration, Makerere University, Kampala, Uganda; ^8^ Katholieke Universiteit (KU) Leuven Department of Rehabilitation Sciences, Katholieke Universiteit (KU) Leuven, Leuven, Belgium; ^9^ University Psychiatric Centre (UPC) Katholieke Universiteit (KU) Leuven, Leuven, Belgium

**Keywords:** exercise, physical activity, sport, suicide, migration, forced displacement

## Abstract

**Introduction:**

Displaced youth face numerous stressors and are at high risk of experiencing suicidal ideation. Sport-for-protection programs offer a potentially effective strategy to reduce this risk; however, randomized controlled trials (RCTs) evaluating interventions in displacement contexts are lacking.

**Methods:**

Displaced youth and youth from host communities in five humanitarian settings across Uganda, aged 15 to 24 years, with at least mild symptoms of anxiety and/or depression and suicidal ideation, were randomized to a 13-session sport-for-protection program or a wait-list control. The Generalized Anxiety Disorder-7 (GAD-7) and Patient Health Questionnaire-9, adolescent version (PHQ-9-A) were assessed pre- and post-intervention. Logistic and linear regression modeling were conducted.

**Results:**

In total, 542 of 817 young people (66.3%) reported suicidal ideation (PHQ-9-A ≥1) in the past 2 weeks. Of these 542, 277 were randomized to the experimental group and 265 to the control group. The median age was 19.0 years, 45.6% were boys, 13.1% had a self-reported or observed disability, 25.8% were from host communities, and 74.2% were displaced youth. The prevalence of suicidal ideation dropped to 11.5% following the sport-for-protection intervention, compared to 53.2% in the control group (Cohen’s h = 0.94, P<0.001, indicating a large difference; odds ratio for no suicidal ideation following sport-for-protection vs the control condition = 8.7, 95% confidence interval = 5.6-13.5).

**Conclusion:**

Sport-for-protection is an effective stand-alone or adjunctive intervention to reduce suicidal ideation among young people affected by displacement in humanitarian settings.

## 1 Introduction

Globally, suicide is a leading cause of death among young people (1), with vulnerable populations such as displaced youth at particularly high risk (2, 3). Displaced populations, including refugees and internally displaced people, experience unique stressors that heighten their risk (4). For example, in Uganda, which hosts the largest refugee population in Africa, displaced youth face numerous stressors, including loss of family, disruption of education, and exposure to violence (5, 6). Research indicates that not only displaced youth, but also their peers in host communities face significant mental health risks (7). Factors such as social and economic pressures, restricted access to resources, cultural conflicts, and disrupted social networks contribute to high levels of psychological distress among both displaced youth and their peers in host communities (8). The trauma related to displacement or witnessing displacement, coupled with the strain on local infrastructure and services, can exacerbate the mental health challenges for both groups. Recognizing the shared experiences of these populations underscores the need for integrated support systems and tailored interventions that cater to the diverse needs of all youth in these environments.

Addressing the needs of youth with disabilities in humanitarian settings is particularly important, as displacement often exacerbates their vulnerabilities and creates additional obstacles to accessing essential services (9). By incorporating disability considerations into program planning, practitioners can promote inclusivity and ensure equitable access, thereby enhancing the mental health and social integration of displaced youth. Additionally, it is essential to integrate gender considerations into these interventions. Boys and girls encounter distinct challenges and barriers that can affect their mental health, suicide risk, and access to support services, with girls often facing a higher risk (7), including in Uganda (10). For instance, young girls in humanitarian settings frequently fall victim to gender-based violence and have reduced access to education due to traditional cultural roles (11). Addressing gender-specific needs ensures that interventions are inclusive and equitable, and improve mental health outcomes and access to resources for all young people, regardless of their gender.

Current evidence for traditional mental health and psychosocial support interventions including pharmacotherapy and psychotherapy within humanitarian settings is limited for both boys and girls. For example, in children and adolescents living in humanitarian settings, to date, no significant difference has been found between psychosocial support interventions and control conditions in improving mental well-being and prosocial behaviour (12). Existing mental health and psychosocial support interventions often face significant limitations in humanitarian settings due to limited available resources and logistical challenges (13). Pharmacotherapy, for instance, often requires a consistent supply of medication and regular follow-up, which can be challenging in settings with disrupted healthcare infrastructure and intermittent access to essential medications. Psychotherapy, while effective in many contexts, also faces obstacles in humanitarian settings. Cultural differences can affect the reception and effectiveness of standard therapeutic approaches, which are often based on Western models of mental health (13). Additionally, the availability of trained mental health professionals is frequently limited in these regions, making it difficult to provide consistent and widespread psychotherapy services.

These limitations necessitate the exploration of other approaches to mental health support in such contexts. Physical activity (PA), defined as “people moving, acting, and performing within culturally specific spaces and context” (14), has emerged as a promising intervention for improving mental health outcomes. Recent trials in adolescents (15) suggest that PA can reduce symptoms of depression and anxiety, which are often precursors to suicidal ideation. PA is inherently flexible and can be adapted to different cultural contexts and resource levels, making it particularly suitable for humanitarian settings. In particular sport-for-protection programs which use sport as a tool to achieve protection outcomes for participants, including social inclusion, social cohesion, and psychosocial well-being (16), are promising and can be implemented with relatively minimal resources. In a recent randomized controlled trial (RCT), we demonstrated the efficacy of sport-for-protection in reducing symptoms of depression and anxiety among displaced youth and their peers from host communities, in those with and without a disability, and in both boys and girls (17).

However, there is currently a lack of research specifically examining the impact of mental health-informed PA-based programs, such as sport-for-protection, on the high burden of suicidal ideation among displaced youth and their peers from host communities. Such research is important as there is limited evidence of the effectiveness of suicide prevention strategies within humanitarian contexts (18). This secondary analysis of our RCT aims to fill this gap by investigating the efficacy of a 13-session sport-for-protection program in reducing suicidal ideation among displaced youth and youth from host communities in Uganda. First, we assessed baseline levels of suicidal ideation among displaced youth and youth from host communities. Second, we evaluated changes in suicidal ideation post-intervention compared to a wait-list control condition. We hypothesized that sport-for-protection will reduce levels of suicidal ideation compared to the wait-list condition. The anticipated findings of this study will contribute to the development of more targeted suicide prevention interventions and inform policy decisions in Ugandan humanitarian settings and similar contexts elsewhere. By exploring alternative interventions such as sport-for-protection, this research seeks to address the critical need for effective, culturally appropriate mental health strategies in resource-limited, humanitarian settings.

## 2 Methods

### 2.1 Participants and procedure

This is a secondary analysis of the Game Connect trial (17) which evaluated a structured sport-for-protection program consisting of 13 sessions over a 16-week period in Uganda,. The program was conducted between September 2022 to July 2023 in five Ugandan districts (Kampala, Adjumani, Kikuube, Lamwo, and Kamwenge). The trial has been registered in ClinicalTrials.gov (Identifier NCT06464627). Sample size, recruitment and randomization procedures have been described elsewhere (17). Details of the intervention are presented in the **Supplementary Material**. Eligible participants in Game Connect were: (a) young displaced or host-community adolescents and young adults aged 15 to 24 years, with (b) at least mild symptoms of anxiety and/or depression, defined by a Patient Health Questionnaire -9, adolescent version (PHQ-9-A) (19) and/or the Generalized Anxiety Disorder-7 (GAD-7) (20) score of 5 or higher. Those with severe depression (PHQ-9-A≥20) and/or anxiety (GAD-7≥15) in the intervention and control condition were eligible to participate, however they were concurrently referred to local services for specialized support. For this secondary analysis, participants need to have a score of 1 or higher on the PHQ-A item 9 exploring suicidal ideation. Eligible young people were allocated by an independent statistician via a random number generator (randomizer.org) to either a 13-session sport-for-protection intervention delivered over a 16-week period or to 16 weeks in a waiting control condition. This extended timeframe was intentionally designed to accommodate potential disruptions common in humanitarian settings, such as adverse weather conditions, security concerns, logistical challenges, or community events. Such flexibility ensured consistent participation and program fidelity throughout the intervention. All participants completed at baseline and immediately post-intervention the interviewer-administered PHQ-9-A (19) and GAD-7 (20). While the PHQ-9-A has been validated for use within the Ugandan context (21), the GAD-7 has been utilized in studies conducted in Uganda (22, 23) but has not yet undergone formal validation in this specific setting. Age (years), sex (male, female), disability (yes vs. no) and legal status (host vs. displaced) were collected at baseline. Disability refers to any observed or self-reported impairment, whether visual, hearing, speech, physical and/or mental, that affects an individual’s ability to perform activities of daily living. These impairments can range from mild to severe and may impact functions such as mobility, communication, self-care, and social interaction. Ethical approval was obtained from the Makerere University School of Social Sciences Research Ethics Committee (reference number: MAKSSREC 10.2023.602) and the Uganda National Council for Science and Technology (reference number: SS2703ES). Informed written consent was obtained from all participants. Consent was obtained from the caregivers of youths aged 15-17 years.

### 2.2 Statistical analysis

Per-protocol analyses with mixed effect logistic regression modelling to test the effectiveness of the intervention on reducing suicidal ideation, and mixed effect linear regression modelling for PHQ-9 and GAD-7 total scores were conducted. Group (intervention vs. control), time (pre vs. post) and group by time and potential moderating factors including age (years), sex (boys vs. girls), presence of disability (yes vs. no), and status (host vs. community) were added as fixed effects. Participants were included as a random effect to the model. The significance level was set at 0.05. To examine post-intervention differences in prevalence of suicidal ideation Chi square tests were used, i.e. the odds ratio for no longer having suicidal ideation in the intervention vs control condition at post-test was calculated. Cohen d based on the pooled standard deviations was calculated using independent t-tests to estimate the between-group effect sizes for changes in PHQ-9-A and GAD-7 scores with effect sizes equal to or larger than 0.20, 0.50, and 0.80 indicating small, medium, and large effects respectively (24). These statistical analyses were conducted using IBM SPSS version 29.0. We also calculated Cohen’s h using arcsine transformation of proportions in order determine if the post-difference in prevalence of suicidal ideation between the sport-for-protection and control condition is meaningful (24). To this end, Cohen’s h was calculated in Excel using the formula =2*(ASIN(SQRT(p_1_))-ASIN(SQRT(p_2_)), where p_1_ is the proportion with suicidal ideation post-intervention and p_2_ post-control condition. A small difference between the two proportions is h *=* 0.2, a moderate difference is h *=* 0.5, and a large difference is h *=* 0.8 (24).

## 3 Results

From 834 youth affected by displacement participating in the study, 17 scores on the PHQ-9 item 9 were missing either at baseline or at follow-up. The completion rate, i.e. participating in at least 10 of the 13 sessions was 96%. Of 817 having a score at baseline and follow-up on the PHQ-9 item 9, 542 (66.3%) reported suicidal ideation at baseline, 277/415 (66.7%) in the sport-for-protection condition and 265/402 (65.9%) in the wait list control condition (P=0.82). The mean (± SD) baseline score of item 9 of the PHQ-9 was 1.3 ± 1.1. Baseline demographic data of the intervention and control groups with suicidal ideation at baseline are presented in **Table 1**. There were significantly more girls in the control condition compared to boys.

**Table 1:** Baseline demographics of participants with suicidal ideation who completed the study.

Characteristics	Total sample (n=542)	Intervention group (n=277)	Control group (n=265)	P-value*
Age (years)	19.0 (3.7)	19.0 (3.0)	19.0 (6.0)	0.38
Sex [n (%) boys/n (%) girls]	247 (45.6%)/295 (54.4%)	149 (53.7%)/128 (46.3%)	98 (37.0%)/167 (63.0%)	<0.001*
Disability [n (%) yes/n (%) no]	71 (13.1%)/471 (86.9%)	32 (11.6%)/245 (88.4%)	39 (14.7%)/226 (85.3%)	0.17
Legal status [n (%) host vs/n (%) displaced]	140 (25.8%)/402 (74.2%)	72 (26.0%)/205 (74.0%)	68 (25.7%)/197 (74.3%)	0.50

*Significant when P<0.05 using Mann Whitney U tests for age and Chi square tests for nominal data. Age is not normally distributed and therefore presented as median (interquartile range). Nominal data are presented as numbers (percentages).

As can be noticed in **Table 2**, when looking at the effectiveness of the intervention in those reporting suicidal ideation at baseline, the prevalence of suicidal ideation dropped to 11.5% following the sport-for-protection program vs 53.2% in the wait-list control condition. The Cohen’s h effect size was 0.94, demonstrating a large difference in prevalence of suicidal ideation following sport-for-protection vs the wait-list control condition. The odds for no longer reporting suicidal ideation following sport-for-protection vs the wait-list control condition was 8.7 (95%CI= 5.6-13.5).

**Table 2:** Changes in suicidal ideation over time following physical activity versus a wait-list control condition.

Variable	Physical activity intervention (n=277)			Wait-list control condition (n=265)			Odds ratio		Group		Time		Group*Time	
	Pre-test N (%)	Post-test N (%)	Δ	Pre-test N (%)	Post-test N (%)	Δ	OR	95%CI	F	P	F	P	F	P
PHQ-9 item 9 (≥1)	277 (100%)	32 (11.5%)	-88.5%	265 (100%)	141 (53.2%)	-46.8%	8.7	5.6-13.5	55.7	<0.001*	272.6	<0.001*	65.3	<0.001*

*Significant when P<0.05. Δ= difference, ° odds for PHQ-9 item 9 = 0 post-intervention vs control. Sex (boys vs. girls), presence of disability (yes vs. no), and status (host vs. community) were added as fixed effects in the model.

Results of the linear mixed model analyses found a significant difference in PHQ-9 and GAD-7 scores between the intervention and control group (see group-effect in **Table 3**: P<0.001). While there was no difference in baseline PHQ-9 (P=0.33) and GAD-7 (P=0.75) scores between groups, there was a difference post-intervention in PHQ-9 (P<0.001) and GAD-7 (P<0.001) scores between both groups. PHQ-9 and GAD-7 improved significantly over time in both the wait-list control and intervention groups (see time-effect in **Table 3**: P<0.001); however, there was a significant interaction effect indicating differential rates of improvement between groups (see group*time effect, **Table 3**: P<0.001). There was a large anti-depressant and anxiolytic effect for the intervention versus wait-list control condition (Cohen d effect sizes in **Table 3**).There was no significant effect for age (years), sex (boys vs. girls), presence of disability (yes vs. no), and status (host vs. community) (data not presented).

**Table 3:** Changes in depression and anxiety over time in those with suicidal ideation and following physical activity versus a wait-list control condition.

Variable	Physical activity intervention (n=277)			Wait-list control condition (n=265)			Cohen d		Group		Time		Group*Time	
	Pre-test (Mean ± SD)	Post-test (Mean ± SD)	Difference	Pre-test (Mean ± SD)	Post-test (Mean ± SD)	Difference	ES	95%CI	F	P	F	P	F	P
PHQ-9	15.1 ± 5.6	2.4 ± 4.1	-12.7	14.6 ± 5.7	10.5 ± 7.6	-4.1	1.34	1.15-1.52	110.8	<0.001*	551.3	<0.001*	147.1	<0.001*
GAD-7	11.9 ± 4.6	1.8 ± 3.1	-10.1	11.7 ± 4.7	8.8 ± 6.1	-2.9	1.46	1.27-1.65	135.7	<0.001*	512.6	<0.001*	155.5	<0.001*

*Significant when P<0.05. GAD-7 = Generalized Anxiety Disorder-7; PHQ-9-A = Patient Health Questionnaire -9, adolescent version, ES = effect size, SD = standard deviation. Sex (boys vs. girls), presence of disability (yes vs. no), and status (host vs. community) were added as fixed effects in the model.

## 4 Discussion

### 4.1 General findings

The current data demonstrate that suicidal ideation is highly prevalent among displaced youth and youth from host communities within humanitarian settings. Almost 2 out of 3 young people who were eligible, (having at least mild symptoms of anxiety and/or depression at baseline) reported thoughts that they would be better off dead, or thoughts of hurting themselves in some way for at least several days in the past 2 weeks. Our data echoes calls for studies to investigate suicide prevention strategies, and explicitly unpack the individual and synergistic effects of multiple-strategies on suicide-related outcomes within humanitarian settings (18).

Our study demonstrated that participation in a 13-session sport-for-protection program significantly reduces suicidal ideation among both displaced youth and their peers from host communities compared to a wait-list control group. The current findings align with existing research indicating that structured physical activity interventions can alleviate suicidal thoughts, although the evidence is inconsistent (25) and research in high-stress environments has been lacking to date (26). Our results underscore the potential of sport-for-protection initiatives to serve as integral components within comprehensive, multidisciplinary suicide prevention strategies in humanitarian settings.

The Game Connect program fosters life skills such as emotional regulation, conflict resolution, decision-making, and relationship building through structured sports activities. These competencies enable youth to navigate the challenges of displacement and build resilience, contributing to reduced suicidal ideation and promoting a strength-based perspective.It can be hypothesized that the effectiveness of the sport-for-protection program can be attributed to several core elements: it promotes social inclusion, fosters community cohesion, and provides a structured routine (27), factors crucial for improving mental health and mitigating suicidal ideation. These benefits are particularly relevant for displaced youth, who often face isolation and instability due to displacement, and in particular vulnerable subgroups such as those with a disability and in girls. Our study demonstrates the effectiveness in these subgroups at risk. Moreover, the program’s impact on reducing anxiety and depression is likely a significant factor in decreasing suicidal ideation. Our results align with broader literature indicating that regular physical activity is associated with lower levels of depression and anxiety in young people (15), which are commonly precursors to suicidal thoughts. By integrating physical activity with community engagement, sport-for-protection offers a transformative approach for suicide prevention, especially in settings where traditional mental health services may be limited. The practical implications of our findings are substantial. For humanitarian organizations and policymakers, integrating sport-for-protection into multi-component mental health support strategies presents a culturally adaptable solution. It can also enhance adherence to existing mental and psychosocial support services, as reduced anxiety and depression may make youth more receptive to and engaged with available resources, ultimately reducing suicidal ideations.

### 4.2 Limitations

Several limitations should be considered when interpreting our findings. First, although suicidal ideation reported via item 9 of the PHQ-9 is a robust, medium-term predictor of suicide attempts regardless of age (28), it should be noted that the PHQ-9 was designed to screen for depression and assess its severity, and not to assess risk for suicidal ideation. Besides this, the item does not distinguish between suicidal ideation and non-suicidal self-injury, potentially leading to overestimation of suicide risk (29). Second, randomization resulted in some young people from the same households being assigned to different groups, and some control group participants had access to similar programs from other NGOs operating in the area. Additionally, public spaces where the sport-for-protection intervention took place led to exposure of the control group to elements of the intervention. Participants in the treatment group also shared their experiences with siblings and friends in the control group, which may have influenced outcomes. Moreover, participants with severe symptoms in the control group were concurrently referred to specialized mental health services, which may have contributed to less suicidal ideation in the control group. These factors suggest that external influences and indirect exposure to intervention elements could have played a role in the mental health improvements seen in the control group. Furthermore, the large observed effect size (Cohen’s h=0.94) may have been partly inflated by factors such as the Hawthorne effect or social desirability bias, where participants report improvements due to attention or perceived expectations rather than intervention effects alone. Additionally, the absence of sex-based block randomisation led to a significant imbalance in sex distribution between the intervention and control groups, with a higher proportion of girls in the control group. Although the per-protocol analysis did not reveal significant moderating effects of sex on the outcomes, this imbalance may have introduced residual confounding. Finally, our study assessed outcomes related to suicidal ideation immediately post-intervention only, which limits understanding of the program’s long-term impact.

### 4.3 Future research

Our study provides promising evidence for the efficacy of sport-for-protection in reducing suicidal ideation in young people within humanitarian settings, but further research is needed before it can recommended. First, future studies should investigate the long-term impact of sport-for-protection on suicidal ideation and overall mental health. Longitudinal research could determine whether the benefits persist beyond the intervention period and if programs help in preventing future mental health crises. Second, additional research should examine the specific mechanisms through which sport-for-protection reduces suicidal ideation. Understanding how elements such as social inclusion, community engagement, and physical activity contribute individually to mental health improvements could refine the program’s design and implementation. Third, research should focus on adapting sport-for-protection programs to various cultural contexts and settings. Evaluating the cultural appropriateness and effectiveness of different sport activities across diverse populations could enhance the program’s impact globally. Fourth, comparative studies between sport-for-protection and other lower intensity mental health interventions (e.g., community-based lay worker-led psychotherapies or digital mental health tools) could provide insights into the relative effectiveness of different approaches in reducing suicidal ideation and improving mental health. Additionally, future studies should aim to control for variables that may lead to contamination, such as indirect exposure of control participants to intervention elements. Implementing strategies like cluster randomization, assigning entire communities to either the intervention or control group, can minimize cross-group interactions. Regular monitoring through surveys or interviews can also help detect unintended exposure to intervention elements among control participants. Employing statistical adjustments, like contamination-adjusted intention-to-treat analyses, can further account for any residual contamination effects. Moreover, future studies should consider strategies to minimize the potential influence of the Hawthorne effect and social desirability bias, such as incorporating observer-blinded assessments and using validated indirect questioning methods such as the unmatched count technique (30). And finally, further research is needed to tailor sport-for-protection to even better support youth with disabilities and address gender-specific needs. Understanding the barriers and needs of these groups can help ensure the program is fully inclusive and accessible (31).

## 5 Conclusion

The significant reduction in suicidal ideation observed in our study underscores the potential of sport-for-protection as a valuable component of suicide prevention strategies in humanitarian settings. By leveraging the benefits of physical activity and community engagement, this approach provides a practical and culturally adaptable solution to address the mental health needs of both displaced and host community youth. Continued research will be crucial to refine and expand these findings, ensuring that interventions remain effective, inclusive, and responsive to the needs of all youth populations.

## Data Availability

The raw data supporting the conclusions of this article will be made available by the authors, without undue reservation.
