# Supplementary material for: A sport-for-protection program reduces suicidal ideation in youth affected by displacement: a secondary analysis of the Game Connect trial in Uganda
Source: Front Psychiatry. 2025 Jul 4;16:1569793. doi: 10.3389/fpsyt.2025.1569793 (PMC12271119; doi:10.3389/fpsyt.2025.1569793)
Supplement: Supplementary file 1 [file SupplementaryFile1.docx]

**Online supplement 1.** Overview of the 13 life skills targeted in Game Connect

Life skills are abilities for adaptive and positive behavior that enable youth to deal effectively with the demands and challenges of everyday life.

Describe in this way, many skills can be considered to be life skills and the nature and definition of life skills are likely to defer across many different cultures and settings.

**Supplementary Table 1.** Overview of the 13 life skills targeted in Game Connect

| **#** | **Life skill:** | **Definition** | **Activities (see Online supplement 2 for a detailed description of the activities)** |
| --- | --- | --- | --- |
| 1. | Citizenship and accountability | Attitudes and beliefs that contribute to your sense of responsibility to yourself and others. | - Marking game   Key learning: I am aware of my role in my own wellbeing.   - Floater   Key learning: I have a sense of responsibility for myself and others. |
| 2. | Conflict resolution | The ability to interact successfully with others and regulate negative emotions and distress by sharing problem solving and collaborating. | - Assertive, passive, aggressive   Key learning: I can problem solve with others. |
| 3. | Coping with emotions and stress | Coping with emotions involves recognizing emotions in yourself and others being aware of how emotions affect behavior and being able to respond to emotions appropriately. Coping with stress is about recognizing the sources of stress, recognizing how this affects you and acting in ways that help to control your levels of stress. | - Hey you   Key learning: I can clearly express, label and describe my emotions.   - Blow the balloon   Key learning: I can control and manage my own behaviors and reactions. |
| 4. | Decision making and problem solving | Decision-Making helps you deal constructively with decisions about your life. Young people actively make decisions about their actions by assessing the different options and what effects different decisions may have. Similarly, problem solving enables you to deal constructively with problems in your life. | - Strategy breaks   Key learning: I can identify a problem, identify options to address the problem and can evaluate options.   - Running goalie pg.94   Key learning: I can make decisions to reach a common goal. |

**Supplementary Table 1.** Continued

| **#** | **Life skill:** | **Definition** | **Activities** |
| --- | --- | --- | --- |
| 5. | Empathy | The ability to imagine what life is like for another person, even in a situation that you may not be familiar with. | - Seated volley ball/hula-hoop football   Key learning: I acknowledge other’s deferring points of view and thoughts.   - Frozen bean bag   Key learning: I can express empathy when am listening to others. |
| 6. | Goal-setting | The ability to identify something you want to accomplish and making realistic plan to achieve it. | - Goal reach   Key learning: I can make a plan for accomplishing the goal.   - Reaching my goal   Key learning: I can work around obstacles that gate in the way of goals. |
| 7. | Relationship building | Effective relationships require mutual trust. Mutual trust a characteristic in relationships that takes time, energy and a commitment to nurture. To develop mutual trust, youth need to develop skills to respect others, show empathy, know how to communicate and cooperate with others, as well as how to negotiate challenging situations with others. | - Blind fold football   Key learning: I can communicate with others.   - Four goal football   Key learning: I can work with others to achieve a common goal (collaboration). |
| 8. | Respect for similarities and differences | To have due regard for and be able to work effectively with people from a range of social and cultural backgrounds and experiences. | - Life auction game   Key learning: I can appreciate my own and other’s values.   - Stigma attack   Key learning: I understand the effects of stigma and can include others. |
| 9. | Self-awareness | Includes your recognition of yourself, your character, strengths and weaknesses, desires and dislikes. | - Bucket ball   Key learning: I can identify my strengths and skills when working with a team.   - River of life   Key learning: I can develop an awareness of my personal qualities, values and perceptions. |

**Supplementary Table 1.** Continued

| **#** | **Life skill:** | **Definition** | **Activities** |
| --- | --- | --- | --- |
| 10. | Self-confidence | The feeling of trust in your abilities’, qualities and judgement. | - My protective shield pg.154   Key learning: I know my personal accomplishments.   - Cherishing strengths pg.156   Key learning: I have positive sense of self. |
| 11. | Self-control | Your ability to control and regulate yourself. This includes your ability to control impulses, delay gratification, direct and focus attention and regulate emotions and behaviors. | - Playing with limits pg.161   Key learning: I can follow healthy limits.   - Score or not pg.163   Key learning: I can control my impulses. |
| 12. | Self-efficacy | The ability to believe in your capacity to exercise choice and influence personal thoughts and behaviors in order to handle a wider range of tasks of situations. | - One to nil pg.168,   Key learning: I am able to influence events in my life   - Football tennis pg. 171   Key learning: I can make plans and carry them out |
| 13. | Sense of belonging | The feeling of being connected and accepted and accepting others from different social and cultural backgrounds. | - Switching teammates pg.177   Key learning: I feel accepted as a member of the team.   - Team boasters pg.178   Key learning: I can give and receive attention from others. |

**Online supplement 2.** Examples of Game Connect activities

*Reference*:* The United Nations High Commissioner for Refugees, Olympic Refuge Foundation, AVSI Foundation, Right to Play, Uganda Olympic Committee & Youth Sport Uganda. 2022. Game Connect. Facilitator Manual. (Revised version)

**Marking game**

*How to play?*

- Set up a playing field with a goal along each end. Both goals have goalkeepers.
- Players work in pairs, one from each team. Only partners can cut the pass from each other. Partners don’t need to shadow each other all the time, but if one gets the ball, the other will try their best to cut the pass from a distance.
- When the ball goes out of play, players dribble it back onto the pitch instead of using throw-ins and corners.
- If a player cuts the pass from another player who is not their partner, their team accepts a penalty.

*Closing discussion*

- In today’s session, what did you work together with your peers to achieve? What were some things that only you could do for yourself?
- In your life, who is responsible for your wellbeing? (Listen for: yourself and others, e.g. coaches, teachers, family, community) Who are you responsible (accountable) to for taking care of yourself? (possible answers: Yourself, family, community)
- This week, what will you do to make sure that you are taking care of yourself?

*Key messages*

- Take responsibility for your actions because failure to do so affects you and others.
- Accept your mistakes and learn from them.
- It is important to take the roles given to us seriously.

*The facilitator manual can be obtained from the first author upon reasonable request.

**Online supplement 2.** Continued

**Floater**

*Overview*

A modified game where there is an extra player who supports the team that has possession of the ball. This player switches teams depending on who has possession.

*Materials*: ball

*Number of players*: 5 or more

*How to play?*

- Form teams for a regular match (2 vs. 2, 3 vs. 3, 5 vs. 5 etc.).
- Assign one extra player to be the “floater”. The floater will go from team to team, depending on who has possession of the ball. Their goal is to always be an open player ready to receive a pass and always be available to support the team in possession.

*Closing discussion*

- In today’s activities, what actions did you take to demonstrate responsibility towards yourself and towards others?
- In your everyday life, what are other ways that you can take responsibility to serve your family, team or community better?

*Key messages*

- Be observant, know yourself and the environment surrounding you.
- Be accommodative and accept people for who they are.

**Online supplement 2.** Continued

**Assertive, passive, aggressive**

*Overview*

A relay race in which players dribble a ball around obstacles. This activity can be adapted for different drills.

*Materials:* one set of materials for each team: ball, cones or obstacles (6 or more), behaviour cards

*Number of players*: any

*How to play?*

Mark a start line and set up a line of cones for each team in front of the start line.

Discuss with participants

- What do each of these words mean: “passive” / “aggressive” / “assertive”?
- What would passive/aggressive/assertive behaviours look like to you?
- Explain that there are different ways of behaving and we are taught how to behave at an early age. How we behave in each situation af­fects how well or badly things will go. Generally, there are passive, aggressive, and assertive behaviours.

Explain and demonstrate

Form teams and give each team a ball and a set of behaviour cards. Each player will pick up a behaviour

card from their team’s set. At their turn, they will read it aloud to their team and decide if it is “passive”

“aggressive” or “assertive.”

- If it is assertive, they must dribble the ball in and out through the cones and back, using only their dominant foot.
- If it is passive, they must dribble the ball in and out through the cones and back, using only their other (non-dominant) foot.
- If it is aggressive, they must dribble the ball with both feet through the cones and back..

*Optional extension activities*

- Invite volunteers to act a role-play to show the difference between passive, aggressive, and assertive behaviour. Use the same situation for each role-play to make the difference clear.
- Ask each team to role-play a situation for the whole group where one person was aggressive or passive. Replay the situation and invite people to ‘freeze’ the acting and replay the story to show assertive behaviour.

*Considerations*

- Emotional safety: This topic may remind youth of negative experiences (e.g. experiencing aggressive behaviour).
- Throughout the session, pay attention to how participants are feeling. Follow up with participants who may be distressed.

**Online supplement 2.** Continued

**Assertive, passive, aggressive (Continued)**

*Closing discussion*

- Which behaviours were passive? Aggressive? Assertive? How was the way you moved the ball connected to the behaviours on the cards?
- Which type of behaviour do you think is most constructive and useful for everyone involved? Why?
- Do you want to change your own behaviour in any way? Why?

*Key messages*

- Being assertive is the most constructive and useful behavior. However, we can choose to be passive or aggressive according to the prevailing situation
- It is good to understand people’s personality traits-take note of non-verbal communication

**Online supplement 2.** Continued

**Hey you**

*Overview*

A guessing game where players work in teams to communicate emotions using their voice and facial expression.

Materials: None

*Number of players*: Any

*How to play?*

Form 2 teams, each in a line.

Explain and demonstrate that:

- Teams will face away from each other.
- Teams will have 15 seconds to come up with an emotion.
- When you say “Go,” teams will turn around and together yell “Hey, you!” (or any other phrase without a particular meaning) at the same time.
- When they yell “Hey, you,” players will use the tone of voice and facial expression that matches the emotion.
- After each emotion, ask players to take one step back.
- Ask each team to guess what the other team’s emotion is.
- Continue playing until teams are far apart.


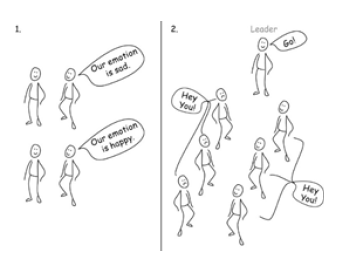


*Considerations*

- Be prepared to discuss why different ways to express emotions are healthy or unhealthy if needed.
- Consider providing information about support services for youth who are dealing with difficult emotions.

*Closing discussion*

- What are some of the emotions that you noticed were expressed in today’s session?
- Why is it important to express our feelings? What happens if we do not?
- What can you do to let others understand how you are feeling?

*Key messages*

- Learn to express your real emotions and do not pretend
- It's good to understand people's emotions before judging them
- Be observant of people's facial expressions and the tones when communicating
- Know how to express your emotions for people to understand you

**Online supplement 2.** Continued

**Blow the balloon**

*Overview*

An exercise where participants imagine anger as a shrinking balloon.

*How to play?*

- Set up a playing field with a goal along each end. Both goals have goalkeepers.
- Discuss with participants that we tend to keep many feelings locked away inside us. When the accumulated load of hurts becomes too much to manage, they burst out like a bicycle tire that has been over pumped. By then, we have no control over them; but if we are more aware of our feelings, we can express them in ways that are safe and do not harm others or ourselves. We do not allow them to build up.
- Blow up a balloon. Hold the end but do not tie it. Ask participants to imagine that the balloon represents anger that has not been expressed and is getting bigger and bigger inside them.
- Ask participants: What would happen if you continued to blow up the balloon? (Listen for: It would burst).
- Say: You were upset, but now you are angry (blow up the balloon more). Now you are really mad (blow more)… You feel like hitting someone/something/shouting (blow up more, the balloon may burst).
- Now, blow another balloon. Tell participants to imagine a situation where they are upset with a friend. The next day you talk to your friend about how upset you were and feel better. (Symbolize this by not blowing the balloon any further, but let it stay the size it was.) As you talk more and more with your friend, the anger goes away (let the air out of the balloon slowly).
- Ask the participants to close their eyes and imagine the balloon. Tell participants: This is your balloon. It is filled with feelings of anger. The feelings are getting bigger and bigger. But you can find someone to talk to, or you tell yourself you are good. Take a deep breath. Relax. The balloon starts getting smaller because air is slowly going out of it. You relax, talk and feel good. The hurt feelings are becoming smaller, eventually they become so small that the balloon becomes limp and is blown away by the wind. Feel the balloon blowing away. Feel light as if a weight has been lifted. Relax and open your eyes. You are feeling so much better.

*Considerations*

Emotional safety

1. Balloons might burst and make a loud sound that could make some participants uncomfortable. Explain this to participants before beginning the activity. Explain that they may opt out of the activity or stop participating at any time if they are uncomfortable. This activity is about anger, which can be a difficult feeling. Pay attention to how participants are feeling. Follow up with participants who may be distressed.

*Closing discussion*

- What sorts of balloons do you have? Full and ready to burst or soft ones?
- When you feel angry, how you calm down? How can others help you?

*Key messages*

- Have self-control to avoid negative reactions
- When faced with problems, open up and talk to someone you trust.
- A problem shared is a problem half solved
- Avoid bottling up anger; find positive ways to cope with it.

**Online supplement 2.** Continued

**Strategy breaks**

*Overview*

A modified game where teams think of ways to win a match using tactics. They can also call on the coach’s support to work out a strategy.

*Materials:* equipment for the sport of your choice

*Number of players:* any

*How to play?*

- During a game, allow each team occasional 3-5 minute breaks to make a strategy that will help them deal with their opponents.
- After letting each team do this on their own the first time, give each team a chance to ask the coach for advice the next time there is a break.
- The coach can give their input but should also ask the players what they think of the coach’s ideas and what their own ideas may be.

*Considerations*

Encourage and support players to identify issues, possible solutions and the pros and cons of different options. Support players, especially those who are shy or less confident, to share their ideas with their team.

*Closing discussion*

- In today’s session, what was a challenge that your team tried to address?
- What options did you think of to address this challenge? How did you decide what to do?
- In your life, what is a decision that you need to make this week? What will you consider in order to identify different options and choose the best one?

*Key messages*

- It is important to understand our challenges, identify, evaluate and apply the most feasible option.
- You can solve your problem(s) when you work with the others as a team.

**Online supplement 2.** Continued

**Running goalie**

Overview

A small-sided game where one player of the opposing team protects one of three set goals, forcing players to score through the other two unprotected goals.

*Materials:* ball, 16 cones (or anything to mark goals and a playing field).

*Number of players*: 2 teams (e.g. 2 teams of 5)

*How to play?*

Set up a playing field with three goals at each end.

Explain and demonstrate that

One player stands behind any of the three goals, but is not allowed to touch the ball. The others play on the field. The player behind the goals can move. The opposing team is not allowed to score into the specific goal s/he is standing behind. Therefore, if a player is about to shoot and score into a specific goal, the fifth player can go behind that goal and the shooter will have to change her/his mind and think about her/his next move.

*Closing discussion*

- In today’s activities, how did your decisions help your team?
- Tell us about a decision you took in your life that helped others.

*Key messages*

- It is important to weigh your options for you to make the right decisions.
- The decisions we make affect us and others within the community in reaching the set goals

**Online supplement 2.** Continued

**Seated volley ball/hula-hoop football**

*Overview*

Players sit on the ground and try to hit the ball over the centre line (volleyball) or remain in a hula hoop and try to score a goal (football).

*Materials*: VOLLEYBALL: ball (lighter and bigger than a volleyball), chalk or anything to mark a centre line, chairs; FOOTBALL: ball, hula hoops or chalk

*Number of players*: 6 or more

*How to play?*

| SEATED VOLLEYBALL:   - Mark a line in the centre of each court (see diagram). - Explain and demonstrate that they will play volleyball, but: - All players must stay seated. - Instead of using a net, players hit the ball over the centre line. - Players are not allowed to stand or use their legs. If they do, the volleyball will be given to the other team and the play continues. | HULA HOOP FOOTBALL:   - Change the size of the playing area to suit the number of players and their passing ability. Lay hula hoops in different areas of the pitch, preferably one for each position. - Assign each player to a hoop. - Explain and demonstrate they will play football, but: - Players cannot leave the hula hoop during the game. - Players cannot stick their hands and arms outside of the hula hoop to intercept or block a pass. - If a pass is missed, the defending team gets possession. |
| --- | --- |

*Closing discussion*

- During the activity, did you try to see things from a teammate’s point of view? If yes, how did this help you? (Possible answer: In seated volleyball, we needed to think about our teammates’ point of view, e.g. what they could/could not do, what they needed.)
- In your everyday life, what are some ways that you respond when someone’s view is different than yours?
- The next time you disagree with someone, what can you to do understand their point of view?

*Key message*

Acknowledge that we have differences in our day-to-day life and everyone should be given an opportunity to share their opinions.

**Online supplement 2.** Continued

**Frozen beanbag**

*Overview*

Players try to balance a ball in the air. If the ball drops, players can ask for help through a role play that highlights barriers to empathy.

*Materials:* balls, empathy blockers cards (see diagram below)

*Number of players*: 4 or more

*How to play?*

- Give each player a ball and an empathy blockers card (see below). Before the game begins, invite players to read their cards and make sure they understand their roles.
- Challenge players to move around the playing area and keep the ball in the air using different body parts. Explain and demonstrate that: If the ball drops, they need to freeze. Another player can ask a frozen player to tell them about their problem (i.e. they’ve lost their ball). As the frozen player talks, the other player should follow the role on their card. (Players do not need to keep their ball in the air while talking.) The other player can then retrieve their ball for them while keeping their own ball in the air.
- After a while, challenge players to keep the ball in the air with a different body part and continue playing.

*Closing discussion*

- What does “empathy” mean?
- When you talked to others in today’s activities, did you feel that they empathized? Why or why not?
- In real life, what are ways that people block empathy?
- This week, what can you do to show empathy when you are listening to others?

*Key messages*

- We should be non-judgmental.
- Show support and encourage fellow youth(s) dealing with stressful emotions.

Empathy blockers cards:

**
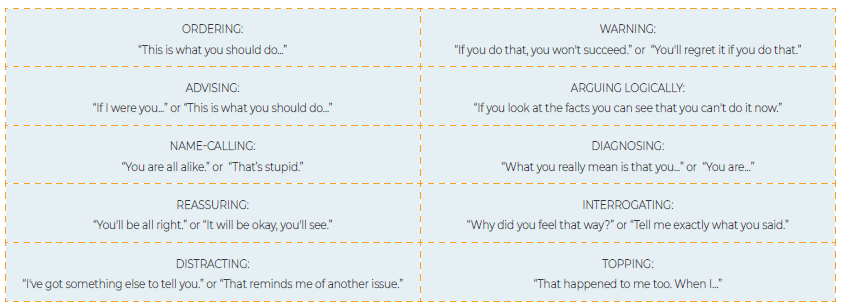
**

**Online supplement 2.** Continued

**Goal reach**

*Overview*

Players work together to reach pieces of paper without crossing a line.

*Materials*: paper and pencils, anything to mark a line on the ground.

*Number of players*: any

*How to play?*

- Mark a start line.
- Invite each player to write/draw 3 goals they have for their lives, and fold their pieces of paper.
- Scatter the papers in the playing area (see diagram).
- Ensure that 2-3 pieces of paper are:
  - within reach of the start line (within a metre);
  - just out of reach (about 1-2 metres away)

very far out of reach (about 3-4 metres away).

- Explain and demonstrate that: The goal of the game is to work as a team to try to gather as many of the goals (pieces of paper) as possible. There are 2 key rules:
  - Players can only touch the ground beyond the start line with their hands.
  - If any player touches the ground with another part of his or her body, the whole team will lose 2 of their goals.
- Give players a few minutes to develop a strategy together and then begin!

*Closing discussion*

- In today’s activities, what strategies did you use to reach your goals? (Possible answer: Players can share the strategies they used to reach the pieces of paper)
- Were you able to reach all of your goals? Why or why not?
- Think about the goals you have for your own life. What are some short-term goals you can create to help you work your way toward your long-term goals?

*Key messages*

- Your goal(s) should be simple and achievable.
- We need to make plans to accomplish the goal.

**Online supplement 2.** Continued

**Reaching my goal**

*Overview*

Players pass through obstacles as they move a ball to the end line. At each obstacle, they have a scenario where they need to make a decision.

*Materials*: flipchart, markers, paper, pencils, chalk (or anything to mark lines on the ground), obstacles (e.g. tables, chairs), ball, chits that say “STOP” and “GO”

*Number of players*: Any

*How to play?*

PART A:

- Introduce the activity by saying that to make our dreams a reality, we have to learn to set goals. Give a few examples like: If I want to pass the exam next week, I have to collect all the notes, study them and revise my lessons this week. This means I cannot go to the market day this week. I must be able to say “no” to my friends.
- Explain that there are some steps that help us set realistic goals. On a flipchart write, “Reaching our goals.” Ask the participants to go through the following three steps individually and write down their thoughts:
- Choose the goal. (Check: Is it realistic? Is it good for you? Who else is this decision good for?)
- Find out what challenges may occur in achieving the goal and the possible solutions to those challenges.
- What are your resources? (Check: Who will help you? Do you have the money? What skills do you have?)

PART B:

- Make a line and place four STOPS along the way. Place an obstacle after each STOP that the participants have to overcome, e.g. an upside down chair or a table. At the end, on the other side of the room, write, “I have reached my goal.”
- Tell the participants that we will try an example together. Invite participants to stand at one end of the playing area. Invite a volunteer to go to the starting point with the ball.
- Place one participant at each STOP. Their role is hold out two chits (face down) for the volunteer to choose.

Explain and demonstrate that

You will share a story of a young man/woman with a goal (see example below). The volunteer will represent this by moving the ball towards the end line. At each STOP, the participant will choose one of the chits.

- If s/he chooses GO: S/he (or someone else) will read out a scenario. S/he then continues with the ball around the obstacle.
- If s/he chooses STOP: S/he (or someone else) will read out a different scenario and then s/he will explain how s/he can move forward.

If the judges are content with the explanation, s/he moves forward to the next stage. If s/he is not convincing enough, his/her turn is over and the judges invite another player to try. There is no going back on a decision that is made.

Select three volunteers (girls and boys) to be the judges. They will assess the choices made and whether the consequences of each choice have been thought through.

After each round, ask the judges to give the ratings and explain why that score was given. Discuss the consequences and the difference that resulted in the life path. Make sure that the participants recognize the connection between what they decide now and what happens later.

**Online supplement 2.** Continued

**Reaching my goal (Continued)**

If time permits, switch roles and do the activity with another story. You can invite participants to create

a story about their own goals. At each STOP, participants can think of a potential obstacle and how to

overcome it.

*Considerations*

- If youth are not comfortable reading/writing, provide alternative ways for youth to do the activity without feeling embarrassed.
- Make sure that before writing down individual goals, participants are supported to reflect on their paths of life, for example, by doing the River of Life activity (see Self-Awareness games), and are supported to identify their hopes and dreams for the next 5-10 years. Young people must be able to each identify their many needs, make a priority list, reflect through the obstacles to achieving the desired goal and decide on a safe decision creatively.

*Closing discussion*

- In the game, which choices were difficult to make? How did you decide what to do?
- In your own life, what are some obstacles that you have faced in reaching your own goals? How did you deal with them?
- Think of a goal you have now. What is an obstacle that you are facing (or might face in the future)? What can you do about it?

*Key messages*

• There are many different ways to achieve a goal.

• We need to routinely reflect, take action on their goal(s) and evaluate their progress

**Online supplement 2.** Continued

**Blind football**

*Overview*

A modified game where all players, except for the goalkeepers, wear a blindfold.

*Materials*: blindfolds (or players can close their eyes).

*Number of players*: 2 teams (e.g. 2 teams of 5)

*How to play?*

- Form 2 teams. All participants, except for the goalkeepers, wear a blindfold. Each player will have one support player who is not blindfolded. The support player cannot touch the ball. Their job is to help instruct their teammates to play the game and get a goal.
- This game should not go longer than 10 minutes before the support player switches roles with their partner who was blindfolded.

*Considerations*

- Emotional safety: Players who are not comfortable wearing the blindfold can just close their eyes. Players who are not comfortable playing with their eyes closed/blindfolded can choose not to participate in this role.
- Physical safety: Caution players to avoid collisions. For example, you might eliminate tackling from the game and require pairs to be a certain distance apart. Allow guides to block balls that may injure their blindfolded partners.

*Closing discussion*

- In what ways did you and your partner/teammates communicate well today? In what ways could your communication improve?
- How did communication help you do your tasks effectively? How did poor communication impact you?
- What can you do this week to communicate more effectively with others in your everyday life? (Consider both verbal and non-verbal communication.)

*Key messages*

- Effective communication builds a trusting relationship.
- We can use both verbal and non-verbal communication skills to relate with others.

**Online supplement 2.** Continued

**Four goal football**

*Overview*

A game of football with four goalposts. Each team works together to score goals on the opposing team while defending their own goals.

*Materials*: 2 balls, cones (or anything to mark goals and a playing field)

*Number of players*: 4 or more

*How to play?*

- Mark off clear boundaries for a square playing area and four clear goals (see diagram).
- Explain and demonstrate that: This game is similar to football with a few major differences:
  - There are four goals and two balls. (Note: If you are playing with a small number of players, use just one ball).
  - There are no goalkeepers.
  - Each team must work together to defend two goals as well as try to score goals on the opposing team.
- After playing for 3-4 minutes, pause the game and give each team time to discuss a strategy.
- Continue playing for 3-4 minute intervals and provide more opportunities for teams to meet and work on improving their strategies for success.

*Closing discussion*

- In today's activities, how did your and your peers decide what plan or strategy to use?
- Did you have any disagreements? How did you handle them?
- In your everyday life, what are some situations where you need to work together with others to reach a common goal?
- When you work together with others this week, what are some things you can do in order to work effectively as a team.

*Key messages*

- Working with others, helps us achieve a common goal.
- It’s important to make plans in life, it helps to set realistic and achievable goals.
- Accepting other people’s opinions, helps the team to easily achieve the goal.

**Online supplement 2.** Continued

**Life auction game**

*Overview*

A game where participants choose to “buy” things that they believe will make them happy in life.

*Materials*: Play money (5 x 1000 Ug Shs for each participant), paper and markers to make picture cards (see steps 1-2 and the example auction) *Play money should preferably look like real money.

*Number of players*: any

*How to play?*

- Ask the participants about what they generally value in life. Encourage them to mention material things as well as abstract things. For example: a good car, house, having children, having a good friend, honesty, a healthy life, to be famous, to be loved and so on. Write these on the cards, and stick them on the wall.
- Select a few from each set and create picture cards. (Make twice as many cards as the number of participants.) Make sure you select both tangible and intangible items.
- Tell the participants that there will now be an auction of these selected items, and each of them can buy whatever they want. Distribute play money of 5000 Uganda shillings (5 x 1000 Ug Shs) to each participant. Tell the participants that in the game, this money will help them to “buy happiness”.
- Explain and demonstrate that: At each auction, two items will go up for sale. A participant can buy one or both items. The participant may also choose not to buy anything. Once the goods are sold, they cannot be returned. Participants are free to buy or save their money.
- Select 10 pairs of items, putting a different value on each. As a rough guide, the sum of the value of all the items should be worth double of what each participant has (or 5000 Ug Shs). This requires the participants to choose. You can even decide to put higher prices on the necessary or valued items, whigach can be abstract. Make sure you have several copies of the items going up for sale, so that you can give them to participants who decide to buy the items.
- One example auction is shown below. Some strategies for conducting the auction are in italics. Make sure you use them in the appropriate place no matter what items you choose to auction. After the auction, facilitate a brief discussion on participants’ perception of how our “wants” are determined by our Value System.
- Start with questions such as:
  - Were there similarities or differences between your choices and your friends’ choices? If so, why?
  - What do you think you were basing your choices on?
  - What do you think influenced the choices of other people, especially those who chose differently from you?
- Our value system is shaped by our family, society and life experiences. It is important for participants to understand that our values make us act in a certain way to meet our “wants,” i.e., our values influence our behaviour. If we critically examine and review our val­ues, then we have the power to change some behaviour that may be harmful for us.

*Closing discussion*

- In the game, how did you decide whether to buy an item? What values were your decisions based on?
- In everyday life, how can knowing someone’s values help you to understand their behaviour and decisions? How do you think this will impact the way you interact with them?
- This week, what is something you can do to learn more about someone else’s values

**Online supplement 2.** Continued

**Life auction game (Continued)**

*Coach’s key message*

- Values and beliefs influence behaviour: For example, if your value lies in physical beauty you may find yourself making certain decisions based on face value. Ask participants to think of other examples where your values and beliefs actually influence the way you behave.
  - Beliefs: Assumptions and convictions that are held to be true by an individual or a group about events, concepts, people and things. Beliefs are the assumptions we make about ourselves, about others in the world and about how we expect things to be. Beliefs are about how we think things really are. Beliefs tend to be deep set and our values stem from our beliefs.
  - Values: Are those things that really matter to each of us/*things we rate highly*. They can be ideas, thoughts, items, beliefs we hold as special. Caring for others, for example, is a value; so is the freedom to express our opinions. Most of us learned our values – or morals, if you prefer – at home, at church or at school.
  - People’s values are different; they cannot be divided into right or wrong categories. Our values are shaped by many factors including age, environment and experiences. A particular person’s values are not permanent. They can change over time as the person ages, changes environment or experiences changes. While each one of us is influenced by our family values, in some cases, children do not share the same values as their parents.

*Key messages*

- It’s important to respect other’s values, beliefs, choices, and preferences.
- Our values and beliefs influence our decision and choices.
- It’s important to critically think and analyze our choices in life.

**Online supplement 2.** Continued

**Stigma attack**

*Overview*

Players dribble around obstacles to get to the “Attack Stigma Post”.

*Materials*: One set of materials for each team: ball, 6-8 cones (or other obstacles), if needed: other sports equipment for dribbling.

*Number of players*: 3 or more

*How to play?*

- Before the game, facilitate a discussion about stigma:
  - What is stigma? (Listen for: When our community believes someone or a group of people is worse than everyone else because of something they are going through, something they have done or one of their characteristics)
  - What are the effects of stigma?
  - What is an example of a way you can attack (or stop) stigma of certain groups in your community? (Possible answers: Being kind in the way you talk about someone; treat everyone with respect and encourage peers, younger siblings etc. to do the same; inviting someone who others do not want to be friends with to join your activities; choose someone who is usually excluded when forming teams in sports or at school).
- Form teams and place 6–8 evenly spaced cones (about 1 metre apart) in a line in front of each group. The last point in the line is called the Attack Stigma Post.
- Explain and demonstrate that: In this relay, players dribble a ball around the markers to the Attack Stigma Post and back. When they reach the Attack Stigma Post, they will tell you one way to attack (or stop) stigma.
- Give teams a few minutes to discuss their ideas for how to attack (or stop) stigma. Remind them that they must be ready to tell you their Attack Stigma idea at the end of the obstacle course. Each team member must have a different idea.

NOTE: Ask prompting questions to support youth to think of more examples if needed.

The relay begins when you say “Attack”.

*Considerations*

- It is important for youth to recognize the effects of stigma. If needed, spend more time discussing this with examples. Focus on positive actions that youth can take and the supports that are available to them.
- Emotional safety: Do this activity in a respectful environment where youth feel comfortable to share. The topic of stigma can bring up different emotions and memories. Tell participants that the topic that they just discussed may have felt difficult. If they feel upset anytime after the activity, they can talk to you or someone they trust for support (share the contact information for specific organizations or helplines). Check how participants are feeling at the end of the activity and close the session with a positive activity.
- Personal safety: In some communities, others may disagree with people who try to challenge stigma. If participants suggest ideas that may put them at risk, support them to understand the risks and to think of appropriate solutions or alternatives.
- Social Inclusion: Be ready to share information about local organizations and resources that support youth facing stigma and discrimina­tion. If you need support identifying appropriate resources, please contact the Game Connect partner that you are working with.

**Online supplement 2.** Continued

**Stigma attack (Continued)**

*Closing discussion*

- What were some new ideas you heard about how to attack (or stop) stigma?
- What is one thing you can do this week to attack (or stop) stigma in your home or community?

NOTE: In some communities, others may disagree with people who try to challenge stigma. If participants suggest ideas that may put them at risk, support them to understand the risks and to think of appropriate solutions or alternatives.

*Key messages*

- Create awareness to stop stigma in the community.
- Stigma affects our self-esteem and social wellbeing.

**Online supplement 2.** Continued

**Bucket ball**

*Overview*

A team game in which teammates depend on each other to place a ball in a bucket.

*Materials*: Ball, 2 buckets or open containers (larger than the ball)

*Number of players*: 4 or more

*How to play?*

- Mark a rectangular playing area (see diagram). Form 2 teams. Ask for one volunteer from each team to be the goalie. Give each goalie a bucket. ( If you have less than 6 players, play without goalies).
- Explain and demonstrate that:
- The objective is to throw the ball into the bucket.
- The goalie on each team will stand behind the end line. The goalie is allowed to move anywhere behind the end line but not over it.
- Team A begins with the ball. Team A will attempt to keep possession of the ball by passing it to each other without dropping it.
- Players not allowed to take any steps when they have the ball in their possession.
- Team A will try to get the ball to a teammate close enough to the bucket to throw the ball in the bucket.
- Team B will try to get possession of the ball. However, Team B members must stay a step away from any player with the ball.

- Possession of the ball is awarded to the other team if: the ball is dropped, or the ball is intercepted or knocked out of the air by the opponents, or the ball leaves the playing area.
- Possession remains with the same team if the other team attempts to catch the ball and drops it. If the defensive team actively knocks it out of the air, it is their ball.
- Players are not allowed to reach over the end line to place the ball in the bucket.

*Other ways to play: Modify a basketball game*

- Place a cone on each foul line.
- Explain and demonstrate that:
- Players A1 and B1 each stand with one foot touching the team cone. A1 and B1 each hold a bucket and can move the bucket to catch teammates’ shot attempts.
- Players try to score into the bucket held by their teammate.
- Basketball rules apply. Exception: A score counts only if the bucket holder has a foot touching the team cone.

**Online supplement 2.** Continued

**Bucket ball (Continued)**

*Considerations*

Ensure all players have opportunities to participate and contribute. If needed, prompt players to communicate and work together.

*Closing discussion*

- In today’s activities, what did you do to help your team to be successful?
- In your life, what are some other situations where you work in a team/group? What sort of skills or strengths do you bring to these teams/groups? (Possible answers: Listening, sharing ideas, teamwork, helping others, sport/artistic skills, writing)
- Why is it helpful to know what your skills and strengths are?
- This week, what is one skill/strength that you can use to do something together with others?

*Key messages*

- It’s important to identify one’s strengths and skills.
- It’s important to be patient and focused.
- It’s good to work in a team to achieve the set target.

**Online supplement 2.** Continued

**River of life**

*Overview*

Participants illustrate events throughout their lives.

*Materials*: Flipchart paper, markers/pens/pencils and rubbers.

*Number of players*: any

*How to play?*

- Invite participants to form pairs. Ask them to discuss the memories of the happy times and the difficult times in their lives if they are comfortable.
- Invite participants to draw their “River of Life”. Ibegins when the participant is born. When there is a happy event, the river flows upwards. When something sad happens, it flows downwards. When an event is neither happy nor sad, the river flows in a straight line.
- Encourage the participants to remember as far back as they can and write or draw the events. The events could be marriages, deaths, entering school, meeting your best friend, learning how to cycle, going on a trip for the first time, or anything else that they feel is important.
- Encourage participants to add key events, qualities and values that they feel have shaped who they are today.
- Encourage each participant to imagine some happy events in the next five years and then 10 years from now and draw them using a different marker.
- Invite participants to share their River of Life with the rest of the group (or a partner/small group) if they are comfortable

*Considerations*

Emotional safety: This is a sensitive activity and may remind youth of negative experiences. If the participant has had traumatic events in life and does not wish to share with a partner or the rest of the group, his/her wishes must be respected. Throughout the session, pay attention to how participants are feeling. Ensure that individual participants are supported during and after this activity. Life is characterized by ups and downs and young people need to be helped to understand that it is only normal. The low points do not have to override one’s life. Through relationships, activities and spirituality, one can move from the down moments to the higher grounds of one’s journey of life. Tell youth that they can talk to you or a trusted adult for support (share the contact information for specific organizations or helplines).

*Closing discussion*

- What did you feel when you analyzed your life? Was it helpful? Why or why not?
- In your life, how did you react to the challenges? What helped you get through the challenge(s)?
- Have you noticed any changes in yourself over your life?
- Why is it important to reflect on your own life experience?
- How can your values and qualities help you reach your goals in the future? How can your values and qualities help you get through chal­lenges in the future?

Key messages:

- It’s important to know that challenges are part of life.
- It’s good to know that our life experience makes us stronger.

**Online supplement 2.** Continued

**My protective shield**

*Overview*

Players create a shield that shows their strengths and accomplishments.

*Materials*: paper, scissors, markers/pens/pencils

*Number of players*: any

*How to play?*

- Spend some time reminding participants about the self-awareness activities that they have done so far.
- Show an example shield (made of paper/cardboard) and discuss with participants what shields are used for (Listen for: To protect, to look after the person holding it).
- Invite participants to create their own shields. They can:
  - Write their names in the center
  - Look at their paper plates and write the best things they like about themselves on one half of the shield. They can also add things they have done that they are proud of.

NOTE: If youth have difficulty thinking of ideas, use prompting questions. Choose or create questions according to the age and ex­periences of the youth and what is valued in their culture(s). Some example questions are:

- What is a new skill you’ve learned?
- Did you ever study really hard and get a good mark?
- Did you work hard to train for a competition before? How did your efforts show in the competition?
- Did you ever help with a community project?
- Do you know how to cook? / Are you good at art? Etc.
- Do you have a talent that nobody knows about?
- Ask the participants what this shield means to them and how it would help them. (Possible answers: “To protect myself when others are mean to me” or “to show the world what a wonderful person I am” or “no one can hurt me now”).
- Invite each participant to share his/her shield with the group.
- Consider inviting participants to put up the shields on the wall so that they can look at them whenever they come to the group. If that is not possible, another option is to keep the shields and display them during the next session. A display of these plates during the life skills sessions is a powerful reminder for the participants of their strengths.

*Closing discussion*

- What are some things that you've worked hard to do? How have you seen yourself grow?
- When you feel discouraged, how can it be helpful to think about these accomplishments?
- How can knowing about your skills and unique qualities help you make the right choices in life?

*Key messages*

- Know yourself and be comfortable with it.
- Explore your strengths, and use them to help people around you.
- Everybody in life has something they can contribute to the community.
- Knowing your limitations helps you deal with them.

**Online supplement 2.** Continued

**Cherishing strengths**

*Overview*

Players complete tasks in four stations. Different stations allow players with different strengths to excel.

*Materials*: jump ropes, hoops, small piece of wood/stone, 2 timers, anything to use as a scoreboard. You can change the tasks and use different materials.

*Number of players*: any

*How to play?*

- Set up 4 stations (see below) and form teams. Explain that each team will rotate through 4 stations (approx. 5 min. per station), you can change their tasks at each station. :
  - Station A – Speed: Players run to the opposite side of the field, one at a time. Calculate the score by totalling the best times of each individual.
  - Station B – Power: Depending on the size of the teams, decide on the number of players that will do each task:
    - Players jump the rope as many times as they can.
    - Players do as many push-ups as they can.
    - Players spin the hoop around their hips as many times as they can.
    - Calculate the score by totaling the numbers of all team members.
  - Station C – Intellect: Within 2 minutes, players write as many sport-related words as possible. Players share their lists and each word that is unique (not written by any other player) gets one point. If literacy levels are lower, change the task or have players work together.
  - Station D – Persistence: Players balance on one foot on a small piece of wood (or stone). The time of the best result is noted down.
- Ask teams to keep score.

*Closing discussion*

- Which skills were obvious during the exercises and which were less so, or not obvious at all?
- How did players look for a way to use their own skills to achieve the assignment?
- What was the role of the other team members in players’ finding their own skills?
- Why is it important in real life to cherish your strengths? What is something you can do this week to cherish your strengths?

*Key messages*

- Cherishing your strengths helps you to contribute positively to the community.
- Other people trusting you helps you build self-esteem and self-confidence.
- Be open minded to identify new strengths.

**Online supplement 2.** Continued

**One to nil**

Overview

A small-sided game where players need to maintain a difference of one point between teams.

Materials: equipment for the sport of your choice *Choose a sport where players play attack and defense.

Number of players: 2 teams

*How to play?*

- Set up a playing field with a goal along each end. Both goals have goalkeepers.
- Explain that teams can win by only one goal: When a team scores a goal, it cannot score another goal. When it wins back the ball, it tries to keep possession and stop the other team from scoring. If the other team scores to equalize, then both teams can try to score to make it 2–1. If a team scores a goal to make it 2–0, both goals are disallowed and the score returns to 0–0.
- At the end of the game, the team that is ahead by one goal is the winner. If neither team is ahead by one goal, the game is a draw.

*Closing discussion*

- What strategies did you use to control the score in the game?
- In your everyday life, what actions and decisions do you have control over?
- This week, what action or decision you can take to help reach your short or long-term goals/hopes?

*Key message*

- Acknowledge what you can and cannot control to make the right decisions.
- Make choices in order to influence events in your life.

**Online supplement 2.** Continued

**Football tennis**

*Overview*

A small-sided game that combines rules from football, tennis and volleyball.

*Materials:* Ball, 8 cones (or anything to mark the playing area).

*Number of players*: Any (adjust the playing area for the number of players).

*How to play?*

- Mark off a playing area like a tennis court, with side-lines, end lines and a centre line. Invite players to imagine a waist-high net running across the centre line from one side of the court to the other.
- Form teams and invite them to take positions on opposite sides of the centre line.
- Explain and demonstrate that the rules are a combination of football, tennis and volleyball:
  - Players may not touch the ball with their hands or arms.
  - One player serves from behind the end line, volleying the ball over the net.
  - The players on the receiving team must return the ball over the net. They have a maximum of 3 touches. The ball can hit the ground only once on their own side of the net.
  - Play continues until one team fails to return the ball in bounds.
  - The ball is “out” if it touches the ground outside the boundary lines.
  - Servers continue serving until they lose serve.
  - Players keep score in simple points or as in tennis or volleyball.
- Pause at various points of the game and invite teams to discuss and create their own strategies.

Players/coaches can modify the game by changing the:

- Number of bounces and / or touches allowed and kind of touches allowed, e.g. headers only
- Number of players who must touch the ball before a return
- Height of the net.

*Closing discussion*

- What are some plans or strategies that your team made in today’s session? How did you work together to put your plans into action?
- In your everyday life, what are some plans that you can make to help you to be healthy and happy?
- What concrete steps can you take to carry them out?

Key messages

- Make plans and execute them to completion.
- Reflect on your plans and improve on them.
- Sharing your plans with others helps you improve and achieve them.

**Online supplement 2.** Continued

**Switching teammates**

Overview

A modified game where teams are changed throughout the match.

Materials: equipment for the sport of your choice.

Number of players: 2 teams

*How to play?*

Play a game between two teams. Keep score but change the membership of each team regularly (e.g. every 5 or 10 minutes), so that players switch from being opponents to teammates and back again throughout the match.

*Closing discussion*

- In today’s activities, what did others do to help you feel welcome on a team? How did you help others feel welcomed into your team?
- In your everyday life, what are some communities (groups) where you feel you belong?
- This week, what is one thing you can do to help someone else feel connected or accepted?

*Key messages*

- Acceptance in any environment starts with you.
- Have positive attitude toward others.
- Change is a fact of life, embrace it.

**Online supplement 2.** Continued

**Team boosters**

*Overview*

A modified game with a modified scoring system that includes points for making positive statements to one another.

*Materials*: equipment for the sport of your choice, pencil and pad/paper (or anything to track points).

*Number of players*: 2 teams

*How to play?*

- Play a regular match but adjust the way points are given. For example, for a basketball match: 1 basket = 1 point; 1 free throw = 1 point; 7 passes = 1 point; 1 block = 1 point; 1 steal = 1 point; Getting 1 rebound before it hits the ground = 1 point and 1 shot off = 0.5 points.

Be ready to keep track of a lot of points!

NOTE: Adjust how many passes or rebounds etc. it will take to equal one point depending on the sport and ability of the participants.

- Also encourage participants to make genuine positive comments to each other and keep a tally of how many each side says. At the end of the game, award points to teams for making 3 or more positive statements to each other.

*Closing discussion*

- What were some ways that your peers encouraged you in today’s session? What were some ways that you tried to encourage others?
- In your everyday life, what was a time when someone showed you that they cared? How did that impact you?
- This week, what are some things we can do in our group to care for one another?

*Key messages*

- Appreciate others in order to be appreciated.
- Appreciating others motivates and fosters friendship.

**Online supplement 3.** Background and overview of the Game Connect program

***S1. Background***

In 2020, the Olympic Refuge Foundation (ORF) initiated Game Connect, a Sport-for-protection (SfP) program in Uganda with a budget of USD 1,500,000 implemented over 36 months, from August 2020 to August 2023.  The current randomized controlled trial lasted from September 2022 to July 2023.

The program was implemented by a consortium led by the AVSI Foundation in collaboration with Right to Play, Youth Sport Uganda, the Uganda Olympic Committee (UOC), and the United Nations High Commissioner for Refugees (UNHCR), with support from the ORF.  The program sought to enhance the mental health and psychosocial well-being of young people (aged 15-24) in both the refugee and host communities through sport-based interventions intentionally designed to promote socio-emotional and life-skills development among young people, while also strengthening their social and support networks and access to support services.

The geographical scope of the program encompassed Kampala and the refugee settlements of Adjumani in Adjumani district, Kyangwali in Kikuube district, Palabek in Lamwo district and Rwamwanja in Kamwenge as well as the surrounding host communities. AVSI retained oversight and provided backbone support to the program, while implementation was driven by different partners in specific locations (Right to Play in Adjumani, Youth Sport Uganda in Kampala and AVSI in Kamwenge, Kikuube, Lamwo).

Program activities centred around delivery of a 16 week structured sport-for-protection curriculum. This was supplemented with a package of wrap around supports and community engagements (inter-community events and festivals and peaceful co-existence activities; community sensitisation around mental health issues and the role of sport; service delivery mapping and establishment of referral pathways; care giver support through champion parents; personal support by coaches for participants at household level through visits and a peer-support approach).

***S2. Sport-for-protection curriculum***

Development of the curriculum (facilitator guide) was led by Right to Play, supported by the consortium partners to ensure contextual relevance. The curriculum has 63 sport-based activities/games organized into 13 categories of life skills. The curriculum was reviewed after one year of implementation based on feedback from young participants, parents and caregivers, coaches and stakeholders.

***S3. Training of trainers***

A Training of Trainer (ToT) model was deployed to support the roll-out of Game Connect in the five locations. Consortium partner technical staff and district sports officers participated in a five-day TOT and later cascaded the training to field level implementers (coaches). The training purposed to provide and understanding of the sport-for-protection approach, the specific activities of curriculum, and develop basic training and facilitation skills. Twenty six participants (13 females and 13 males) were trained, including four District Sports Officers. A further 5 day ToT refresher was delivered to consortium partner staff in year two, i.e. 28 staff (10 females and 18 males), again with district representatives in attendance.

***S4. Recruitment and training of coaches***

Game Connect coaches were recruited from within program operating locations. The Office of the Prime Minister, UNHCR, and district stakeholders supported the recruitment process. Coaches required an educational background in social work or experience in leading sport, sport-for-protection or other youth activities, as well as basic English literacy skills (reading and writing) and ability to speak the native and/or other spoken languages in their area of operation. Attempts were made to ensure recruitment of male and female coaches and well as those from both refugee and host community backgrounds. Over the course of program implementation, 83 coaches were active across the five locations with 59 active coaches at any point in time.

To improve competences for efficient program delivery, coaches initially participated in an eight day training workshop delivered in their area of operation. The training covered the theory and practice of the sport-for-protection curriculum (see: <https://www.unhcr.org/media/sport-protection-toolkit>). Training in safeguarding, physical and psychological first aid, and Olympic Values was also provided. Additionally, coaches received complementary trainings such as facilitation and communication skills basics in mental health, a sports administrative course and monitoring and evaluation. Settlement protection and Mental Health and Psychosocial Support (MHPSS) service providers were included in the training to support the embedding of Game Connect within the local humanitarian response. Each coach received ongoing mentoring, supervision and support from the program manager and local programme coordinators. Operating procedures incorporated peer-to-peer coach support; establishment of a community of practice to share learning across regions; and coach self-care.

To promote sustainability, coaches identified peer leaders from within their groups who had shown dedication, leadership skills, interest and knowledge of the sport and life-skills during the sport-for-protection sessions. Over the course of the program, 161 peer leaders were identified across the various sites (109 refugees and 52 host community young people). Peer leaders were also trained alongside coaches during refresher trainings.

***S5. Sport-for-protection activities***

The program teams identified safe spaces for SfP activities in each community. Over 100 spaces were identified and support (human resource and small amounts of financial resource) provided to clear, enhance and maintain the spaces as necessary. Evaluation criteria for safe spaces included proximity to settlements, accessibility to youth residences, the presence of water, sanitation, and hygiene (WASH) facilities, participant perceptions of the venue, ownership or access rights.  UOC coordinated the procurement of necessary sports kit and equipment.

Game Connect coaches assessed eligible youth (those within the target age bracket, residing in the target communities). Young people identified as having at least mild symptoms of anxiety and/or depression were eligible to engage in the program. Those experiencing severe symptoms were also referred for specialized clinical care (some were retained within the programme while this was ongoing).

Within the entire Game Connect program (August 2020 to August 2023), 12.706 youths were enrolled in the entire Game Connect program across the five refugee hosting districts of whom 30% were nationals, 70% were refugees, and 508 (4%) were young people with a disability. Fifty-one percent of the enrolled participants were males and 49% female, while 56% were in school and 44% out of school.

Upon completion of the enrolment, coaches assigned young people to groups comprising an average of 25. Coaches were assigned groups with a coach to youth group ratio of 1:4. Groups were formed based on key unifiers such as age (15 to 19 and 20 to 24), location proximity and other characteristics for example young mothers, in and out of school young people. Additionally, grouping was inclusive with a mix of males and females and youth with disability.

Coaches agreed with young people the safe space to be used, and the appropriate time to conduct their weekly sport-for-protection activities. Overall, coaches formed 487 groups comprising 12,293 enrolled young people. Participation in at least 10 of the 13 sport and life skills sessions was considered as having completed the program. The overall completion rate for young people who engaged was 96%.

**S6. Wrap around support and community engagement**

In addition to the 13 sport-for-protection sessions over a period of 16 weeks, the consortium organized two annual events (the first in Kampala and the second in Lamwo) bringing together young people from the different locations.  These events lasted for one day with a goal of improving social cohesion, inclusion, and psychosocial wellbeing for the refugee and host community youth, fostering peaceful co-existence within the communities. Specific objectives of these annual evens were to: (a) increase girls and women’s participation in sport, and (b)  improve social cohesion among the refugee and host community youth. Each implementing settlement would bring two mixed football and netball teams respectively, with each team comprising of both females and males and at least one person with disabilities from the Game connect direct participants. Olympic values of fair play and mutual respect for opponents, while playing/striving to perform at one's best (joy of effort) was the central Olympic Value. Champion parents and community leaders were awarded.

Besides these annual events, at community level, 4.622 community members (parents/caregivers and community leaders) were mobilized by coaches throughout the three years to attend sensitization sessions to enhance knowledge on mental health, mental illness, signs and symptoms of mental illness, and how to manage these. Sensitizations were conducted in collaboration with local mental health partners (e.g., ALIGHT, Strong Minds, and TPO Uganda) in the areas of implementation.

 To facilitate access to critical services, the programme team mapped and established contact with MHPSS and other service providers in all locations. Over the course of the program implementation, 558 young participants were referred for specialized and non-specialised MHPSS services, education, or health services (among others). Game Connect coaches made household visits to 11.699 of the young participants (8.157 refugees, 3.542 nationals). Through home visits, coaches were able to follow up with those who were missing SfP sessions and help reduce barriers to access, support with off-field protection issues, interface with parents and caregivers and provide referrals to other services for unmet needs.
